# Tuning the Composition and Structure of Amorphous Molybdenum Sulfide/Carbon Black Nanocomposites by Radiation Technique for Highly Efficient Hydrogen Evolution

**DOI:** 10.1038/s41598-017-16015-y

**Published:** 2017-11-22

**Authors:** Pengfei Cao, Jing Peng, Siqi Liu, Yu Cui, Yang Hu, Bo Chen, Jiuqiang Li, Maolin Zhai

**Affiliations:** Beijing National Laboratory for Molecular Sciences, Radiochemistry and Radiation Chemistry Key Laboratory of Fundamental Science, the Key Laboratory of Polymer Chemistry and Physics of the Ministry of Education, College of Chemistry and Molecular Engineering, Peking University, Beijing, 100871 China

## Abstract

Amorphous molybdenum sulfide/carbon black (MoS_x_/C) nanocomposites are synthesized by a facile one-step γ-ray radiation induced reduction process. Amorphous MoS_x_ shows better intrinsic activity than crystalline MoS_2_. And the composition and amorphous structure of MoS_x_ could be expediently tuned by absorbed dose for excellent catalytic activity. Meanwhile, the addition of carbon black leads to a significant decrease of charge transfer resistance and increase of active sites of MoS_x_/C composite. Consequently, MoS_x_/C nanocomposite shows Pt-like catalytic activity towards hydrogen evolution reaction (HER), which requires an onset over potential of 40 mV and over potential of 76 mV to achieve a current density of 10 mA cm^−2^, and the corresponding Tafel slope is 48 mV decade^−1^. After 6000 CV cycles, the catalytic activity of MoS_x_/C shows no obvious decrease. However, when platinum (Pt) foil is used as counter electrode, MoS_x_/C composite show better catalytic activity abnormally after long-term cycling tests. The dissolution of Pt was observed in HER and the Pt dissolution mechanism is elucidated by further analyzing the surface composition of after-cycling electrodes, which offers highly valuable guidelines for using Pt electrode in HER.

## Introduction

Hydrogen, because of its advantages of environmental friendliness and high energy density, has been considered as an ideal energy candidate for the sustainable development^1,2^. However, now most of the hydrogen is produced by steam-reforming reaction and chlor-alkali industry. Electrochemical production of hydrogen from the splitting of water by the hydrogen evolution reaction (HER) has long been considered as a highly promising way to produce hydrogen on a large scale^3^. Pt-group metals are considered as the most effective HER catalysts. However, they are not suitable for large-scale application due to their high cost and low abundance, so novel catalysts with low cost, earth abundance, high catalytic activity and stability in strong acids are needed for HER^4,5^. Recently, transition-metal-based materials, such as transition metal nitrides^6^, transition metal oxides and hydroxides^7^, transition-metal dichalcogenides^8^ have been widespread reported as new competent electrocatalysts for water splitting. For example, molybdenum sulfide nanoparticles, including crystalline molybdenum disulfide (MoS_2_)^9,10^ and amorphous molybdenum sulfide (MoS_x_)^11–13^, have become a promising alternative for their potential to meet these demands.

Crystalline MoS_2_ has a typical transition-metal dichalcogenide (MX_2_) layered sandwich structure. The edge planes of crystalline MoS_2_ are active sites for HER, whereas their basal planes are chemically inert^14^. Benefitting from widely research, three universal strategies have been suggested to improve the HER activity of crystalline MoS_2_-based catalysts^15^: (1) increasing the density of active sites; (2) enhancing the intrinsic catalytic activity; (3) improving the conductivity and diffusion properties of the MoS_2_ materials. During the past decade, significant advances have been achieved on crystalline MoS_2_-based catalysts^16,17^. However, compared with widely researched crystalline MoS_2_, amorphous MoS_x_ does not have a certain composition and structure, and the x changes between 2 and 3. To date, the structure of amorphous MoS_x_ has been proposed to be polymeric^12^. Yeo and co-workers^18^ studied the active sites of amorphous MoS_x_. Lee and co-workers^19^ investigated chemical and phase evolution of amorphous MoS_x_ during the HER process. Nevertheless, it is difficult to identify the detailed catalytic mechanism of amorphous MoS_x_ for its variable composition and structure. Even so, the universal strategies mentioned above still can be used to guide our research. Meanwhile, amorphous MoS_x_ can be synthesized under milder conditions, such as wet chemical process^20^ and electrodeposition^21^. The amorphous structure and morphology of MoS_x_ can offer more active sites and higher intrinsic activity, which may enhance the catalytic activity towards HER^22^.

However, the low conductivity of amorphous MoS_x_ limits its application as an efficient HER catalyst. Enhancing the electronic transport in MoS_x_ is a key issue to improve its catalytic performance. The introduction of conductive supports in MoS_x_ can reduce the aggregation of MoS_x_ nanosheets, as well as provide fast electron transfer channels. Carbon materials, owing to their high conductivity, earth abundant, large specific surface areas and high stability, have been regarded as ideal supports for electrocatalytic materials. Carbon materials such as porous carbon^23^, carbon nanotube^24^, carbon fibers^25^, as well as graphene^26^ have been investigated as supports of MoS_x_. Recently, we found that highly conductive carbon black (CB) can enhance the catalytic activity of amorphous MoS_2_ obviously^27^. With regard to that CB is active and economic, MoS_x_/CB composites would have great potential for commercial application in HER.

Solutions irradiated with γ-ray can produce solvated electrons (*e*_sol_^−^) and other radiolysis products. Solvated electrons are very strong reducing agents which can be used to reduce high valence metal ions^28,29^ and graphene oxide^30^. Moreover, γ-ray reduction exhibits many advantages over other chemical methods, such as environmental friendly procedure, mild conditions, high reduction efficiency and easy scaled-up production. Reduction process induced by γ-ray radiation has been used to synthesize CdS^31,32^ and ZnS^33^. Solutions containing high valence Mo ions and S species irradiated with γ-ray may be used to fabricate MoS_x_. Zhang and co-workers synthesized MoS_2_ powder by a complicated irradiation-hydrothermal-annealing method^34^. Nevertheless, there are no reports about one-step γ-ray radiation synthesis of MoS_x_ for HER till now.

Now most of the reported MoS_x_-based catalysts require an onset over potential of more than 100 mV, which can’t satisfy the requirements of practical application. In our previous work^24^, hydrothermal process was used to synthesize MoS_2_/CB. While in this work, for the first time, the MoS_x_/C nanocomposite was prepared by a facile one-step and easy scaled-up γ-ray radiation reduction process. XPS, XRD and TEM analysis were used for analyzing the composition and structure of MoS_x_. The catalytic performance of MoS_x_/C composite towards HER and the influence of absorbed dose were further investigated. By tuning the composition and structure, MoS_x_/C nanocomposite shows Pt-like catalytic activity towards HER. At the same time, the dissolution of Pt was detected during the HER process, and the dissolution mechanism of Pt is discussed in detail. It is expected that this work provides new approaches to synthesize the amorphous MoS_x_/C catalysts for HER as well as some guidelines for the using of Pt electrode.

## Results and Discussion

Considering the interaction of γ ray with EG can produce solvated electrons^21^, and solvated electrons can reduce MoS_4_^2−^ to MoS_x_ nanoparticles, and in the presence of CB, the as-prepared MoS_x_ nanoparticles will load on the sheets of CB to produce MoS_x_/C composite. Firstly, the influence of preparation conditions on the composition and structure of MoS_x_/C composites are investigated and the results are listed in Table 1.

**Table 1:** Sample abbreviations, corresponding preparation conditions and composition of different materials.

Samples	(NH_4_)_2_MoS_4_ (mg)	CB (mg)	Dose (kGy)	S/Mo		Mo^4+^/Mo^6+^	S_higher_^a^/S_lower_^b^
				ICP-AES	XPS	XPS	XPS
MoS_x_/C-1	20	40	50	2.49	2.45	1.58	1.28
MoS_x_/C-2	20	40	100	2.40	2.36	1.68	1.20
MoS_x_/C-3	20	40	220	2.28	2.25	2.11	1.03
MoS_x_	20	/	100	2.25	2.22	2.20	0.94

^a^S_higher_ represents S atoms of apical S^2−^ and/or bridging S_2_^2^^−^; ^b^S_lower_ represents S atoms of unsaturated S^2−^ and terminal S_2_^2−^.

As shown in Table 1, ICP-AES and XPS analysis show a consistent trend that the S/Mo ratio decreases gradually with the increasing of absorbed dose. Moreover, the S/Mo ratio is between 2.5 and 2.2, indicating the structure of MoS_x_ is neither typically crystalline MoS_2_ nor amorphous polymeric MoS_3_. And at the same absorbed dose, the S/Mo ratio of MoS_x_/C-2 is larger than that of MoS_x_. In the preparing process of MoS_x_/C-2, part of radiation energy from γ-rays was absorbed by CB, the energy absorbed by EG was reduced, and this will reduce the numbers of solvated electrons for reduction of (NH_4_)_2_MoS_4_.

**Figure 1:**
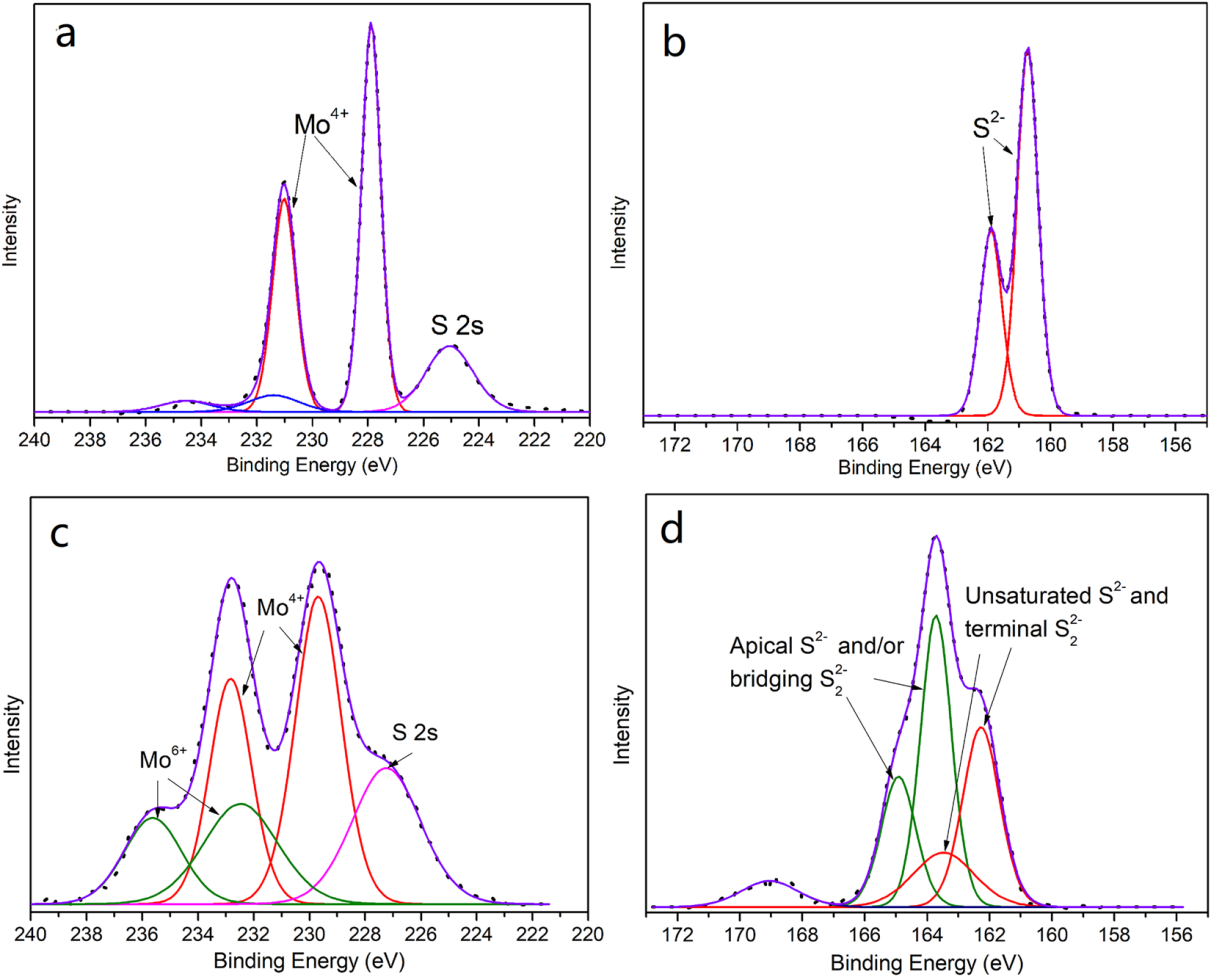
XPS spectrum of pure MoS_2_ powder and MoS_x_/C-2. (**a**) Mo 3d spectrum of pure MoS_2_ powder; (**b**) S 2p spectrum of pure MoS_2_ powder; (**c**) Mo 3d spectrum of MoS_x_/C-2; (**d**) S 2p spectrum of MoS_x_/C-2.

To further explore the composition and chemical states of both Mo and S in MoS_x_/C nanocomposites, XPS data was carefully analyzed. Pure MoS_2_ powder was used as a reference material. Figure 1a and b show the Mo 3d and S 2p spectrum of pure MoS_2_ powder, respectively. As shown in Fig. 1a, the doublet peaks located at 232.1 eV and 228.9 eV correspond to characteristic peaks of Mo^4+^ 3d_3/2_ and 3d_5/2_ of MoS_2_, respectively. A S 2 s peak is observed at about 226.0 eV. The doublet peaks located at higher binding energy may be caused by the slight oxidation of MoS_2_. And in Fig. 1b, the doublet peaks at 163.1 eV and 161.9 eV are assigned to the S^2−^ 2p_3/2_ and 2p_1/2_ of MoS_2_, respectively. However, the chemical states of MoS_x_ synthesized by γ-ray radiation are much more complicated. All the radiation synthesized MoS_x_ materials show similar XPS spectrum. Figure 1c and d show the typical Mo 3d and S 2p spectrum of MoS_x_/C-2, respectively. As shown in Fig. 1c, besides the doublet peaks at lower binding energy (232.5 eV and 229.3 eV) which belong to Mo^4+^ of MoS_2_, the doublet peaks at higher binding energy of 235.9 eV and 232.7 eV can be attributed to unreduced Mo^6+^, This result indicates the structure of MoS_x_/C is different with that of hydrothermal synthesized MoS_2_/CB in our previous work^24^. And in Fig. 1d, the S 2p spectrum shows no obvious spin-orbit splitting, indicating the bonding states of S atoms in MoS_x_/C-2 are complex. Herein, our result was analyzed according to an analysis process proposed by Hu and coworkers^35^. The energy difference between S 2p_3/2_ and 2p_1/2_ was set as 1.18 eV for data fitting. The doublet peaks at binding energy of 163.5 eV and 162.3 eV can be attributed to the unsaturated S^2−^ and terminal S_2_^2−^ 2p_1/2_ and 2p_3/2_ of MoS_2_, respectively. The doublet peaks at higher binding energy of 165.0 eV and 163.8 eV can be assigned to apical S^2−^ and/or bridging S_2_^2−^ 2p_1/2_ and 2p_3/2_ of MoS_x_, respectively. According to Yeo and co-workers’ research^18^, S atoms with higher binding energy should be the catalytic active sites. The ratios of different types of Mo and S atoms are calculated and listed in Table 1. As shown in Table 1, the ratio of Mo^4+^/Mo^6+^ increases with the increasing of absorbed dose. This is due to that the Mo^6+^ ions were reduced by the solvated electrons and higher absorbed dose would produce more solvated electrons. This result is in consistent with the trend of S/Mo atomic ratio. With the increasing percentages of low valence Mo^4+^ ions, the S/Mo ratio decreases gradually and becomes closer to that of MoS_2_, which means the reduction degree of MoS_4_^2−^ increases with the increasing of absorbed dose. Meanwhile, the ratio of S_higher_/S_lower_ shows an opposite tendency with the increasing of absorbed dose.

Powder XRD was used to investigate the structure of as-prepared MoS_x_/C nanocomposites. Figure S1 shows the XRD patterns of CB, MoS_x_ and MoS_x_/C composites. No obvious diffraction peak was found in the XRD spectrum of MoS_x_, which indicates the amorphous structure of MoS_x_ produced by γ-ray radiation. All the MoS_x_/C samples show similar XRD patterns, and no other diffraction peak appears except the (002) and (100) graphitic reflection plane of CB, which indicates that the structure of MoS_x_ maintains amorphous and does not change significantly with the increase of dose.

**Figure 2:**
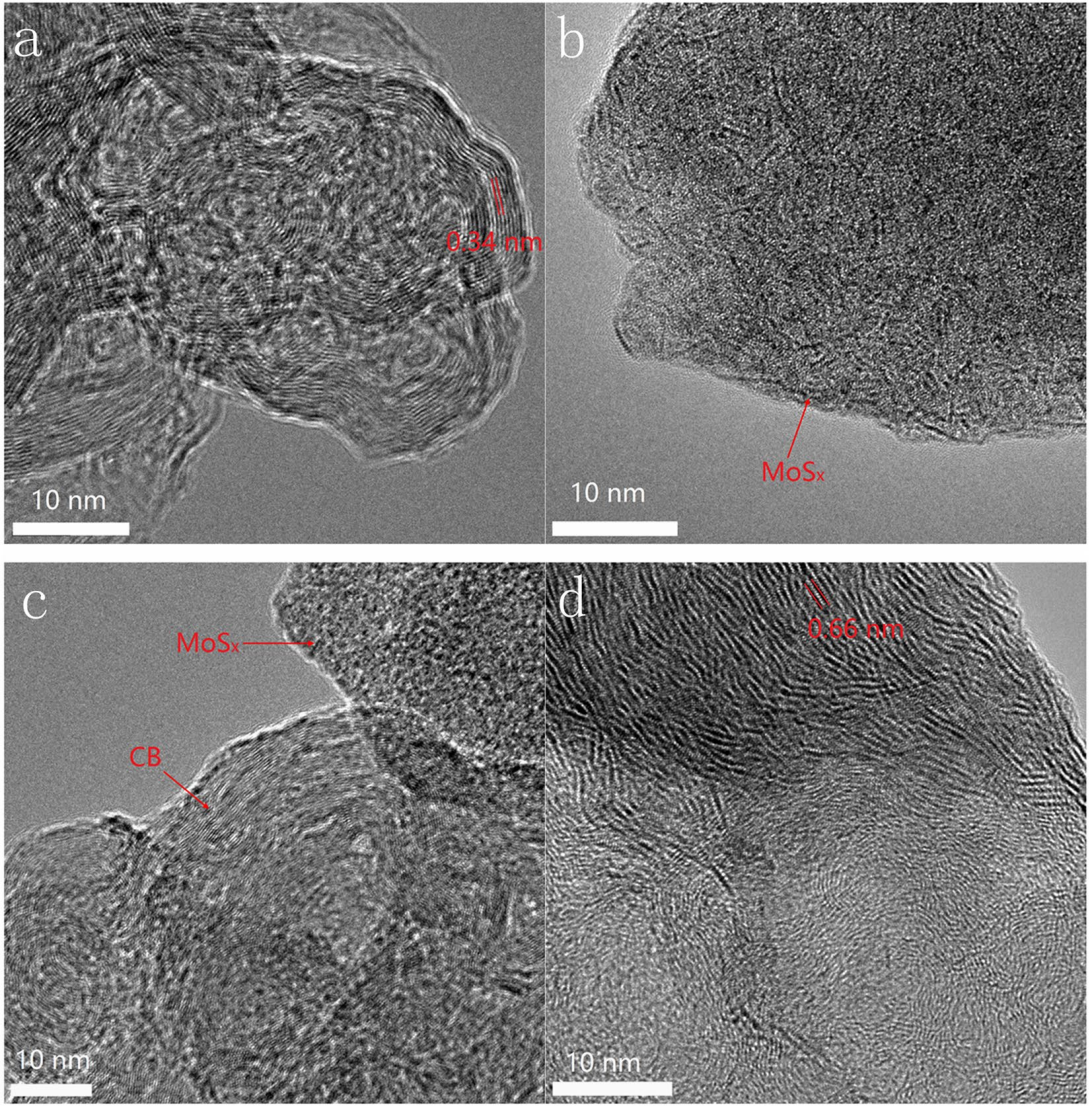
TEM images of (**a**) CB, (**b**) MoS_x_, (**c**) MoS_x_/C-2 and (**d**) annealed MoS_x_/C-2.

TEM amnalysis further demonstrates the morphology and structure of MoS_x_/C nanocomposites. Figure 2a shows the TEM image of CB. The marked inter planar d-spacing of 0.34 nm corresponds to the (002) lattice plane of graphitic CB, which agrees well with the XRD result. However, as shown in Fig. 2b, no ordered structure is observed, indicating the formation of amorphous MoS_x_. Figure 2c shows the image of MoS_x_/C-2, CB and amorphous MoS_x_ can be observed easily, indicating MoS_x_ retains amorphous structure in the nanocomposite. Annealing process is always used to rearrange the atoms and adjust the structure of materials. An annealing process was used to treat MoS_x_/C-2 composite. MoS_x_/C-2 was heated to 350 °C, and maintained at this temperature for 12 hours in the atmosphere of N_2_. As shown in Fig. 2d, after the annealing process, typical two-dimension nanosheets and the layers of MoS_2_ can be observed. This result indicates that annealing process leads to the formation of crystalline MoS_2_ in the nanocomposite. The marked inter planar d-spacing of 0.66 nm corresponds to the (002) lattice plane of MoS_2_, and this spacing is larger than the layer-to-layer spacing of 0.61 nm in bulk MoS_2_, indicating a significant lattice expansion^36^. Moreover, twisted and discontinuous crystal fringes can be observed, indicating the low crystallinity even after the annealing process.

Based on the above analysis, the possible reactions during the process of synthesis are as follows:

1 $${{\rm{HOCH}}}_{{\rm{2}}}{{\rm{CH}}}_{{\rm{2}}}{\rm{OH}}\,\mathop{\longrightarrow }\limits^{{\rm{\gamma }} \mbox{-} {\rm{ray}}}\,{{\rm{e}}}_{{\rm{sol}}}^{-}+{\rm{H}}\cdot {+\mathrm{HOCH}}_{{\rm{2}}}\mathop{{\rm{C}}}\limits^{+}{\rm{HOH}}$$

2 $${{\rm{MoS}}}_{{\rm{4}}}^{{\rm{2}}-}\,\mathop{\longrightarrow }\limits^{{{\rm{e}}}_{{\rm{sol}}}^{-}}\,{{\rm{MoS}}}_{{\rm{x}}}{+{\rm{S}}}^{{\rm{2}}-}$$

**Figure 3:**
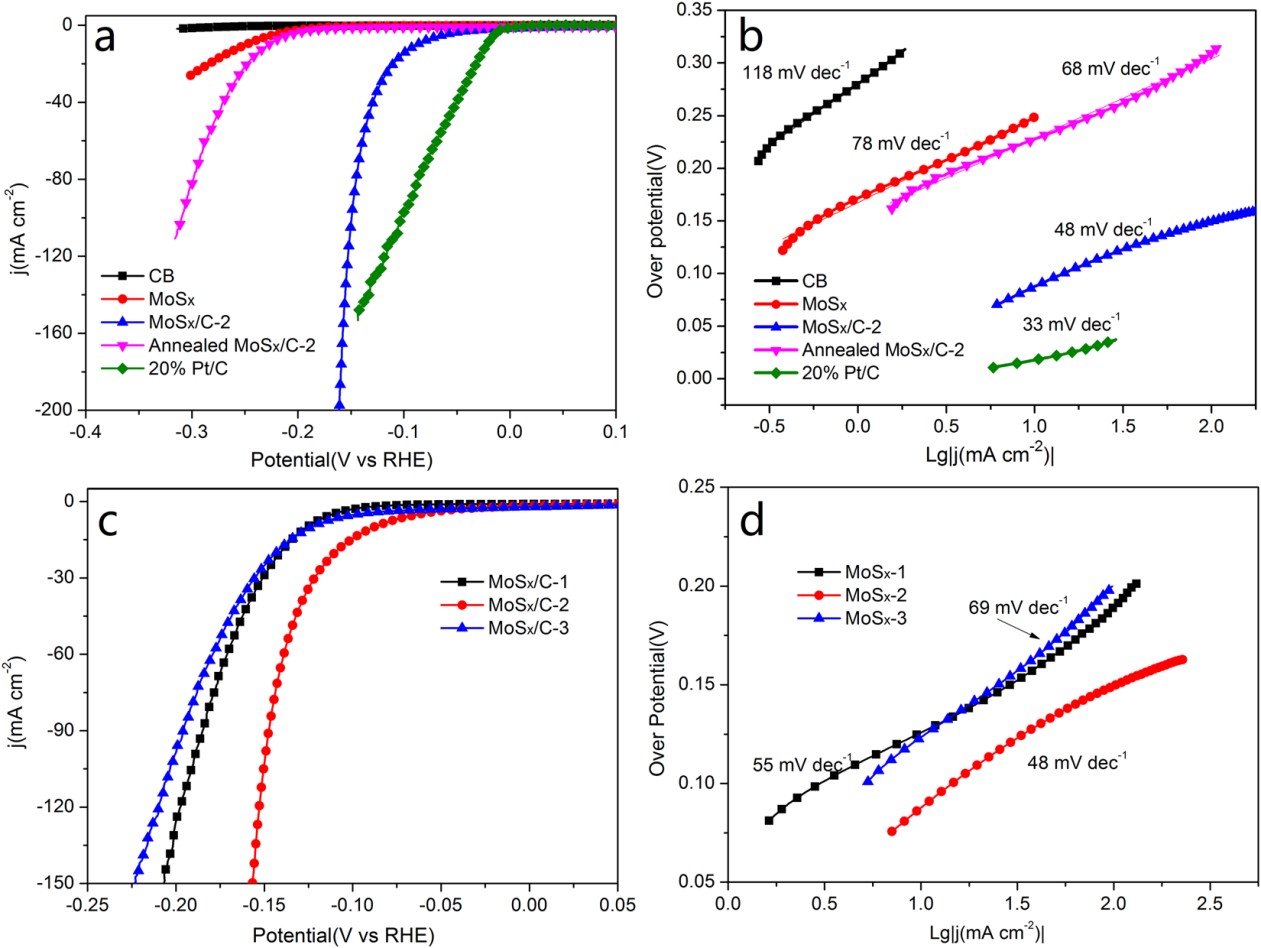
(**a**) Cathodic polarization curves of CB, MoS_x_, MoS_x_/C-2, annealed MoS_x_/C-2 and commercial 20% Pt/C. (**b**) Corresponding Tafel plots of different samples. (**c**) Cathodic polarization curves of MoS_x_/C-1, MoS_x_/C-2, MoS_x_/C-3. (**d**) Corresponding Tafel plots of MoS_x_/C-1, MoS_x_/C-2, MoS_x_/C-3.

To characterize the HER performance of the as-synthesized materials, MoS_x_, all MoS_x_/C nanocomposites and commercial 20% Pt/C were tested in a standard three-electrode system. Figure 3 shows the catalytic activity of different samples. As shown in Fig. 3a, the commercial 20% Pt/C electrocatalyst exhibits the lowest onset over potential of 10 mV and an over potential of 25 mV to achieve a current density of 10 mA cm^−2^. This result is in accordance with previous report^37^. CB is inert to catalyze HER, and amorphous MoS_x_ shows poor HER activity. In contrast, MoS_x_/C-2 exhibits high HER activity, which requires an onset over potential of 40 mV and an over potential of 76 mV to achieve 10 mA cm^−2^. This performance is better than most reported MoS_x_-based catalysts (Table S1) and even close to that of Pt/C catalyst. Furthermore, the current density of MoS_x_/C-2 increases sharply with the increasing of over potential. However, after annealing treatment, the catalytic activity of annealed MoS_x_/C-2 is much lower than initial MoS_x_/C-2, which requires an onset over potential of 150 mV and an overpotential of 206 mV to achieve 10 mA cm^−2^. Compared with MoS_x_ and annealed MoS_x_/C-2, the highly efficient catalytic activity of MoS_x_/C-2 is attributed to the addition of CB and the composition and amorphous structure of MoS_x_.

The mechanism of HER in acidic medium can be summarized to three elementary reactions^37^: Firstly, the Volmer reaction occurs, (Equation 3). After the Volmer reaction, hydrogen may be generated by two different reactions: one is the Heyrovsky reaction, (Equation 4), the other is the Tafel reaction, (Equation 5). So, for an integrated HER process, Volmer-Heyrovsky mechanism or Volmer-Tafel mechanism should be involved.

3 $${{\rm{H}}}_{{\rm{3}}}{{\rm{O}}}^{+}+{{\rm{e}}}^{-}\to {{\rm{H}}}_{{\rm{ads}}}{+{\rm{H}}}_{{\rm{2}}}{\rm{O}}$$

4 $${{\rm{H}}}_{{\rm{ads}}}+{{\rm{H}}}_{{\rm{3}}}{{\rm{O}}}^{+}+{{\rm{e}}}^{-}\to {{\rm{H}}}_{{\rm{2}}}+{{\rm{H}}}_{{\rm{2}}}{\rm{O}}$$

5 $${{\rm{H}}}_{{\rm{ads}}}+{{\rm{H}}}_{{\rm{ads}}}\to {{\rm{H}}}_{{\rm{2}}}$$

6 $$\eta ={\rm{a}}+{\rm{b}}\times \,{\rm{Lg}}|j|$$

The relationship between over potential and current density is shown in Equation 6 (Tafel formula), where η is the over potential, j is the current density, a is a Tafel constant, and b is Tafel slope. Tafel slope is always used to reveal the inherent reaction processes of HER, because it is determined by the rate-limiting step of HER. If the rate-limiting step is Volmer, Heyrovesy or Tafel reaction, the corresponding Tafel slope is about 120, 40, 30 mVdecade^−1^, respectively. Therefore, the polarization curves were presented in Tafel plots to explore the detailed mechanism of HER. As shown in Fig. 3b, the Tafel slope of CB is 118 mV decade^−1^, indicating the rate-limiting step is Volmer reaction. The 20% Pt/C exhibits a value of 33 mV decade^−1^, indicating the Volmer-Tafel mechanism of HER, and this result is consistent with previous research results^4,14^. MoS_x_/C-2 exhibits a small Tafel slope of 48 mV decade^−1^, which corresponds to the Volmer-Heyrovesy mechanism. Whatever, the annealed MoS_x_/C-2 exhibits a much higher Tafel slope of 68 mV decade^−1^. Thus, in the application of MoS_x_/C for HER, the crystalline structure of MoS_x_ is not favor for the catalytic performance.

Since absorbed dose can influence the ratio of S/Mo and the ratios of different types of Mo and S atoms, the catalytic activity of different MoS_x_/C materials were also investigated. As shown in Fig. 3c, for MoS_x_/C-1, MoS_x_/C-2 and MoS_x_/C-3, the onset over potential is 99 mV, 40 mV and 84 mV, respectively, and the over potential of 10 mA cm^−2^ is 126 mV, 76 mV and 124 mV, respectively. MoS_x_/C-2 shows the best catalytic activity among all the MoS_x_/C nanocomposites, and this result is different from Yeo’s report^18^, which reports that catalysts containing higher percentages of S active sites have better catalytic activity. Nevertheless, in our case, MoS_x_/C-1 has the highest percentages of S atoms with higher binding energy, but the catalytic activity of MoS_x_/C -2 is much better than MoS_x_/C -1, and MoS_x_/C-3 shows a similar catalytic activity with MoS_x_/C-1. As shown in Table 1, the variation of reduction degree (the ratio of Mo^4+^/Mo^6+^) and percentages of S atoms with higher binding energy (S_higher_/S_lower_) is in contrast with the absorbed dose. Therefore, the composition and structure of MoS_x_/C composites and their catalytic activity can be tuned by absorbed dose. And for the catalytic activity of MoS_x_/C composites, 100 kGy is an optimal absorbed dose. The Tafel slopes are 55, 48, 69 mV decade^−1^ for MoS_x_/C-1, MoS_x_/C-2 and MoS_x_/C-3, respectively, indicating the catalytic mechanism of all the MoS_x_/C materials is Volmer-Heyrovesy mechanism.

**Figure 4:**
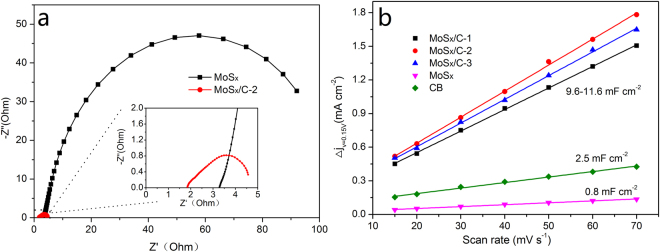
(**a**) Nyquist plots of MoS_x_ and MoS_x_/C-2, inset shows the enlarged EIS spectra; (**b**) Linear fitting for the capacitive currents of CB, MoS_x_ and all the MoS_x_/C nanocomposites versus scan rates.

In order to further investigate the mechanism of the excellent performance of MoS_x_/C nanocomposite in HER, electrochemical impedance spectroscopy (EIS) was performed to study the electrode kinetics. As shown in Fig. 4a, the EIS spectra of MoS_x_ and MoS_x_/C-2 were dominated by a single capacitive semicircle at medium frequency range, suggesting the catalytic reaction was limited by the charge transfer steps. For MoS_x_, the charge transfer resistance (R_CT_) is about 120 Ω, which is obtained by fitting the electrochemical impedance data. And for MoS_x_/C-2, due to the addition of highly conductive CB, the R_CT_ decreases to about 2 Ω. R_CT_ is related to the electrocatalytic kinetics at the catalyst/electrolyte interface, and a lower value corresponds to a faster electron transfer, so the significant decrease of R_CT_ indicates a fast electron transfer and consequently facile HER kinetics at the catalyst/electrolyte interface. These experimental results identify that the strategy of combination MoS_x_ with CB is highly effective to enhance the HER activity, because the presence of CB will lead to rapid electron transfer from the catalyst to the electrode.

Intrinsic activity is another significant factor to assess the property of a catalyst. Exchange current density (j_0_) and per-site intrinsic catalytic activity reflect the inherent catalytic properties of catalysts. j_0_ is obtained by applying extrapolation method to the Tafel plots. As shown in Table 2, for MoS_x_/C catalysts, the j_0_ is 72, 223 and 103 μA cm^−2^ for MoS_x_/C-1, MoS_x_/C-2 and MoS_x_/C-3, respectively. The j_0_ of all the MoS_x_/C catalysts are about 20 times larger than the reported crystalline MoS_2_^38^, and the j_0_ of annealed crystalline MoS_x_/C-2 is only about one-ninth of the initial MoS_x_/C-2, which suggests that the amorphous structure is better than the crystalline structure. With a modest reduction degree and active sites, MoS_x_/C-2 shows the highest intrinsic activity. The j_0_ of MoS_x_ is also much smaller than those of MoS_x_/C catalysts, this can be attributed to the addition of CB. The per-site intrinsic catalytic activity of as-prepared catalysts are further assessed by using the turnover frequency (TOF). TOF represents the number of hydrogen molecules produced per second per active site. We followed an estimation process proposed by Jaramillo and coworkers^20^. The detailed estimation steps have been demonstrated in our previous work^27^.

**Table 2:** HER parameters of various samples.

Samples	j_0_(μA cm^−2^)^a^	C_dl_(mF cm^−2^)	Roughness Factor	TOF(s^−1^)^b^
MoS_x_/C-1	72	9.6	160	0.5
MoS_x_/C-2	223	11.6	194	1.4
MoS_x_/C-3	103	10.6	177	0.4
MoS_x_	28	0.8	14	0.1
Annealed MoS_x_/C-2	25	/	/	/
CB	/	2.5	/	/

^a^j_0_ is obtained from Tafel curves by using extrapolation methods; ^b^TOFs are calculated at the over potential of 150 mV.

The capacitance of the catalyst was estimated with cyclic voltammetry performed at various scan rates in a potential window of 0.05–0.25 V. The representative cyclic voltammograms of MoS_x_/C-2 are shown in Figure S2. The pseudo-rectangular shapes indicate there is no obvious Faradaic current in this potential window. The current density readings at 0.15 V were extracted from the cyclic voltammograms. As shown in Fig. 4b, current density is proportional to the scan rate, indicating a pure non-Faradaic response. The capacitance of the catalyst is half of the slopes. As shown in Table 2, the specific capacitance (C_dl_) of MoS_x_ is only 0.8 mF cm^−2^ while all the MoS_x_/C materials show much larger value of about 11 mF cm^−2^. The enhancement of C_dl_ identifies that the addition of CB can reduce the aggregation of the formed MoS_x_ nanoparticles. Roughness factor was then obtained by using the reported value of 60 μF cm^−2^ for an atomic flat MoS_2_ catalyst. As shown in Table 2, the roughness factor of MoS_x_ is 14, and for MoS_x_/C nanocomposites, the roughness factors are about 13 times larger than the value of MoS_x_. Larger roughness factor indicates more effective active sites, which is beneficial for the HER. Table 2 shows the TOF of MoS_x_ and all the MoS_x_/C nanocomposites at the overpotential of 150 mV. MoS_x_/C-2 shows the highest TOF value of 1.4 H_2_ s^−1^ per active site, which means it has the highest per-site intrinsic catalytic activity, and this result is consistent with previous analysis.

Based on the above analysis, the excellent performance of MoS_x_/C-2 in HER can be attributed to the following two reasons: (1) optimal absorbed dose tunes the composition and structure of MoS_x_; (2) the addition of CB leads to a significant decrease of charge transfer resistance and increase of active sites.

**Figure 5:**
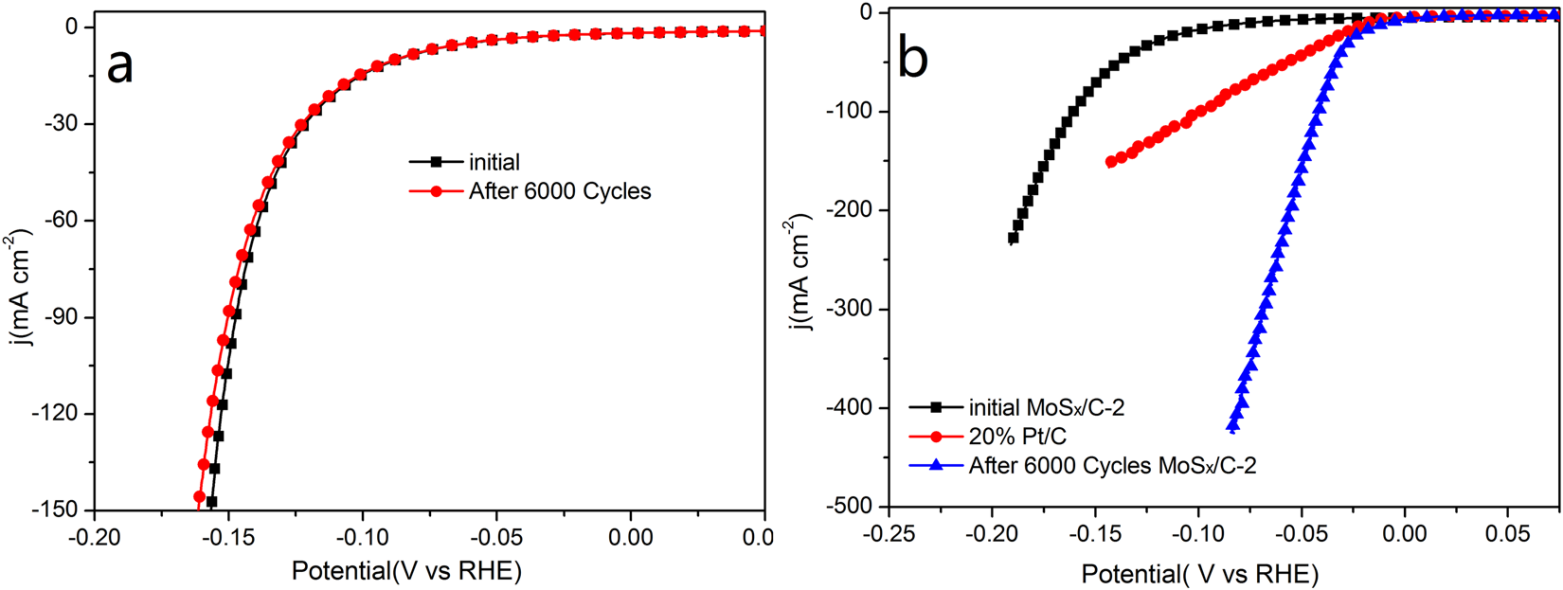
Cathodic polarization curves of MoS_x_/C-2 of initial and after 6000 CV cycle tests. (**a**) Graphite rod as counter electrode; (**b**) Pt foil as counter electrode.

Long-time stability is another significant factor to evaluate a catalyst. Long-term cycling test of representative MoS_x_/C-2 catalyst was measured by CV test. As shown in Fig. 5a, when graphite rod is used as counter electrode, the catalytic activity of MoS_x_/C-2 shows no obvious decrease after 6000 CV cycles, indicating the excellent catalytic stability of MoS_x_/C catalysts during HER process.

However, when Pt foil is used as counter electrode, the catalytic activity of after-cycling MoS_x_/C-2 material is even better than the initial material and 20% Pt/C. As shown in Fig. 5b, after 6000 CV cycles, the after-cycling MoS_x_/C-2 requires an onset over potential of nearly 0 mV and an over potential of only 9 mV to achieve 10 mA cm^−2^. This performance is even much better than commercial 20% Pt/C catalyst, which is recognized as the best catalyst for HER. Furthermore, the Tafel slope of after-cycling MoS_x_/C-2 material reduces to 33 mV decade^−1^, which is similar to the 32 mV decade^−1^ of Pt/C (Figure S3). This result indicates that the catalytic mechanism of after-cycling MoS_x_/C-2 proceeds a Volmer-Tafel mechanism. Moreover, the change of catalytic mechanism indicates that there may be a change of structure and composition in MoS_x_/C-2 during the CV test.

**Figure 6:**
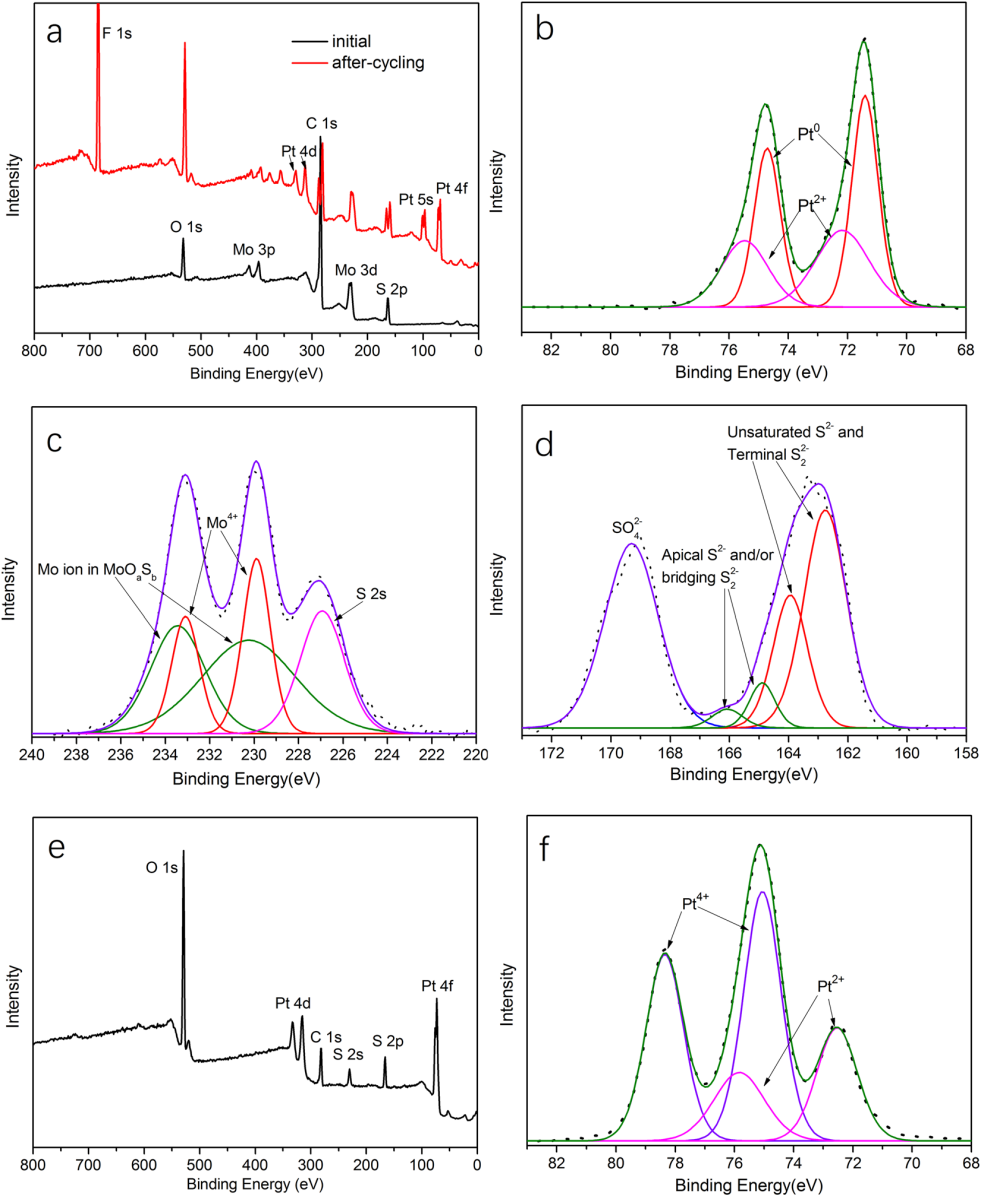
XPS spectrum of different materials. (**a**) XPS spectrum of MoS_x_/C-2 before and after 6000 CV cycles. (**b**) Pt 4 f spectrum of after-cycling MoS_x_/C-2 (**c**) Mo 3d spectrum of after-cycling MoS_x_/C-2; (**d**) S 2p spectrum of after-cycling MoS_x_/C-2. (**e**) XPS spectrum of the golden-colored material formed on the Pt counter electrode during CV tests. (**f**) Pt 4 f spectrum of the golden-colored material.

In order to explain the enhancement of catalytic activity of MoS_x_/C-2, we investigated the structure and composition change of MoS_x_/C-2 during the CV test. XPS and TEM analysis were performed on after-cycling MoS_x_/C-2. Figure 6a shows the XPS spectrum of MoS_x_/C-2 before and after 6000 CV cycles. Compared with initial MoS_x_/C-2, F and Pt elements appear in the after-cycling MoS_x_/C-2. F can be attributed to Nafion membrane we used during the preparation of working electrode, and Pt should come from the Pt counter electrode. Figure 6b shows Pt 4 f spectrum of after-cycling MoS_x_/C-2. The doublet peaks locate at 71.4 eV and 74.7 eV are assigned to metallic Pt^0^ and the doublet peaks locate at higher binding energy (72.4 eV and 75.7 eV) are related to Pt^2+^ ^39^. Figure 6c shows the Mo 3d spectrum of after-cycling MoS_x_/C-2. Compared with initial MoS_x_/C-2, the peaks corresponding to Mo^6+^ disappeared. The doublet peaks at 229.8 eV and 233.0 eV are assigned to the Mo^4+^ ion in MoS_3_, and the doublet peaks at higher binding energy (230.5 eV and 233.7 eV) can be attributed to a Mo ion in molybdenum oxysulfides^35^. This result indicates that high valence Mo ions are reduced during the HER. But due to the addition of Nafion membrane which contains S as well, it is difficult to calculate the S/Mo ratio of after-cycling MoS_x_. Figure 6d shows the S 2p spectrum of after-cycling MoS_x_/C-2, the doublet peaks assigned to apical S^2−^ and/or bridging S_2_^2−^ reduced significantly compared with initial MoS_x_/C-2, and the signal at about 169 eV is attributed to the sulfonic group of Nafion membrane. XPS analysis verifies that the composition and chemical state of MoS_x_/C-2 indeed changed during the CV test. At the same time, Pt counter electrode dissolved during the HER test for some reasons.

Generally, Pt is regarded as a chemical inert and stable material, so it is always used as counter electrode in an electrochemical test system. However, Pt dissolution from the electrode have been found in polymer electrolyte fuel cells (PEFCs)^40,41^. Nevertheless, the potential range applied in PEFCs and operation temperature are higher than those in HER, so there are few reports about Pt dissolution in HER. In 2015, Dong and co-workers^42^ observed Pt dissolution during HER and they speculated the Pt dissolution mechanism. But till now, the detailed mechanism of Pt dissolution during HER is still not very clear, and Pt electrode is still widely used as counter electrode during HER. In this work, the dissolution mechanism of the Pt counter electrode was studied further. Figure S4 shows the photographs of Pt counter before and after CV tests. The surface of Pt foil is covered by golden-colored material after long-time CV test. XPS analysis was then carried out to study the composition and chemical state of the golden-colored material. As shown in Fig. 6e, C, S, O and Pt elements can be observed. S is assigned to adsorbed SO_4_^2−^. The chemical states of Pt were then carefully evaluated by fitting the XPS spectrum of Pt 4 f. As shown in Fig. 6f, the doublet peaks at 72.5 eV and 75.8 eV are assigned to Pt^2+^ and the doublet peaks at higher binding energy (75.1 eV and 78.4 eV) are related to Pt^4+^ ^43^. No signal of metallic Pt^0^ was observed, indicating the chemical states of Pt on the surface of counter electrode are Pt^2+^ and Pt^4+^. So the possible dissolution mechanism is as follows: the oxidizing species formed in the anode reaction can react with Pt foil to generate Pt^2+^ and Pt^4+^, then the oxidized Pt species (Pt^2+^ and Pt^4+^) dissolve in the solution and then migrate to the cathode and are reduced to Pt^2+^ and metallic Pt^0^ at last. There are few reports about the dissolution of Pt during the HER process^42,44,45^ in recent two years, our experimental results confirmed the previous speculation.

Figures S5, S6 and S7a show the TEM and HRTEM images of after-cycling MoS_x_/C-2. As shown in Figure S5, when graphite rod is used as counter electrode, no obvious difference was observed even after 6000 CV cycles. However, the Pt aggregations containing many Pt nanoparticles with a diameter less than 5 nm were observed when Pt foil is used as counter electrode (Figure S6). Figure S7a shows the HRTEM image of Pt nanoparticles, the marked interplanar d-spacing of 0.25 nm corresponds to the (110) lattice plane of Pt nanoparticles, and corresponding EDS spectrum (Figure S7b) confirms the formation of Pt nanoparticles.

The influence of deposited Pt content on the electrode is evaluated as well. Figure S8a shows the first 50 CV cycles of MoS_x_/C-2. With the increasing of cycles, the onset over potential decreases gradually while the current density at the same over potential increases, indicating Pt loading on working electrode increases with the CV tests. Figure S8b shows the polarization curves of MoS_x_/C-2 after different CV cycles. When CV cycles are less than 300, the catalytic activity of MoS_x_/C-2 increases gradually. However, further increase in CV cycles makes no significant difference between 300 cycles and 6000 cycles. ICP-MS analysis shows Pt loadings of MoS_x_/C-2 after 300 cycles and 6000 cycles are 1.5 μg cm^−2^ and 35 μg cm^−2^, respectively. The Pt loading of MoS_x_/C-2 after 300 cycles is much smaller than that after 6000 cycles, but their catalytic activity shows no obvious difference. Higher Pt loading will aggravate the aggregation of Pt nanoparticles, and thereby reduce the utilization efficiency of Pt. The mass activity of MoS_x_/C-2 after 300 cycles is 3.1 × 10^5^ A g^−1^(Pt) at the over potential of 90 mV, which is about 400 times larger than that of 20% Pt/C. Therefore, the improved catalytic performance of after-cycling MoS_x_/C-2 towards HER should be attributed to the deposition of Pt nanoparticles on the working electrode. It is suggested that Pt dissolution should be emphasized for evaluation of the catalyst towards HER when Pt is applied as counter electrode.

## Conclusions

In summary, amorphous MoS_x_/C composites are synthesized successfully by a facile one-step γ-ray radiation reduction process for the first time. The resultant MoS_x_/C shows excellent catalytic activity and cycle stability towards HER, which requires an over potential of 76 mV to achieve a current density of 10 mA cm^−2^, and the corresponding Tafel slope is 48 mV decade^−1^. Amorphous structure of MoS_x_ with suitable reduction degree and active sites and presence of CB support play important roles in the catalytic performance in HER. In addition, the dissolution of Pt was observed during the long-term cycling tests when Pt is used as counter electrode. And the dissolution mechanism is further elucidated by analyzing the surface composition of after-cycling electrode, which is highly valuable for using Pt electrode towards HER.

## Methods

### Materials

Ammonium tetrathiomolybdate ((NH_4_)_2_MoS_4_, 99.95%) was purchased from Acros. Cobot Vulcan XC-72 CB and commercial 20% Pt/C catalyst were purchased from Macklin. Pure molybdenum disulfide (MoS_2_) powder was purchased from Alfa Aesar. Nitric acid (HNO_3_, AR), ethylene glycol (EG, AR) and sulfuric acid (H_2_SO_4_, AR) were purchased from Beijing Chemical Works. Nitrogen gas (99.999%) was purchased from Beijing Haikeyuanchang Practical Gas Co., Ltd. All materials were used as received without further purification.

### Synthesis of MoS_x_/C nanocomposites

MoS_x_/C composites were synthesized by a simply one-step radiation induced reduction process. Typically, 20 mg CB and 40 mg (NH_4_)_2_MoS_4_ were added to 20 mL EG and sonicated for 20 min. Then the mixed solution was saturated with high purity nitrogen gas before the sealing treatment. After that, the suspension was exposed to γ radiation using a ^60^Co source for different doses with a dose rate of 300 Gy min^−1^ at room temperature. After irradiation, the precipitates were separated from the solutions by filtration and washed with pure water and ethanol for several times, and then dried at 40 °C under vacuum. For comparison, the solution of (NH_4_)_2_MoS_4_ without CB was treated according to the above procedure under the same experimental conditions to prepare MoS_x_.

### Composition and Structure Characterization of MoS_x_/C nanocomposites

The inductively coupled plasma atomic emission spectroscopy (ICP-AES) measurements were detected by a Prodigy ICP from Teledyne Leeman Labs. X-ray photoelectron spectroscopy (XPS) measurements were performed on an Axis Ultra X-ray photoelectron spectrometer from Kratos Analytical with an exciting source of Al Kα = 1486.7 eV. The binding energies obtained in the XPS spectral analysis were corrected for specimen charging by referencing C 1 s to 284.8 eV, and Powder X-Ray Diffraction (XRD) was performed on a Philips X’Pert Pro Super diffractometer with Cu Kα radiation (λ = 1.54178 Å). Transmission electron microscopy (TEM) and high-resolution TEM (HRTEM) were carried out on a FEI TECNAI F20 field emission electron microscope at an acceleration voltage of 200 kV. The inductively coupled plasma mass spectrum (ICP-MS) was detected by an ELEMENTAL XR ICP-MS from Thermo Fisher.

### Preparation of Working Electrodes

The carbon paper working electrode was prepared as follows: carbon paper was cut into strips with a width of 5 mm. Then 50 μL catalyst ink was loaded onto the carbon paper strip (area ~0.3 cm^2^, loading ~0.667 mg cm^−2^) and then dried under an infrared lamp.

### Electrochemical Measurements

All the electrochemical tests were performed in a three-electrode system. The details are consistent with the tests we demonstrated in previous work^27^ except that the counter electrode is a platinum foil (~1 cm^2^) or graphite rod (diameter = 5 mm). Linear sweep voltammetry (LSV) and cyclic voltammetry (CV) tests were performed on a CHI 760e electrochemical station. Electrochemical impedance spectroscopy (EIS) was performed on an Autolab PGSTAT302N electrochemical station. All the potential was transferred to reversible hydrogen electrode (RHE) potential by the equation E(RHE) = E(SCE) + 0.260 V. All the polar curves were iR corrected, where R is ohmic resistance obtained by the EIS. Pt dissolution was observed when Pt foil was used as counter electrode. The Pt doped working electrode was then characterized and measured with the same methods.

## Electronic supplementary material

Supplementary data
