# Supplementary material for: Tuning the Composition and Structure of Amorphous Molybdenum Sulfide/Carbon Black Nanocomposites by Radiation Technique for Highly Efficient Hydrogen Evolution
Source: Sci Rep. 2017 Nov 22;7:16048. doi: 10.1038/s41598-017-16015-y (PMC5700052; doi:10.1038/s41598-017-16015-y)
Supplement: Supplementary file 1 — Supplementary data [file 41598_2017_16015_MOESM1_ESM.pdf]

Supplementary data

Tuning the Composition and Structure of Amorphous Molybdenum Sulfide/Carbon Black Composites by Radiation Technique for Efficient Hydrogen Evolution

Pengfei Cao, Jing Peng, Siqi Liu, Yu Cui, Yang Hu, Bo Chen, Jiuqiang Li, Maolin Zhai*

Beijing National Laboratory for Molecular Sciences, Radiochemistry and Radiation Chemistry Key Laboratory of Fundamental Science, the Key Laboratory of Polymer Chemistry and Physics of the Ministry of Education, College of Chemistry and Molecular Engineering, Peking University, Beijing 100871, China

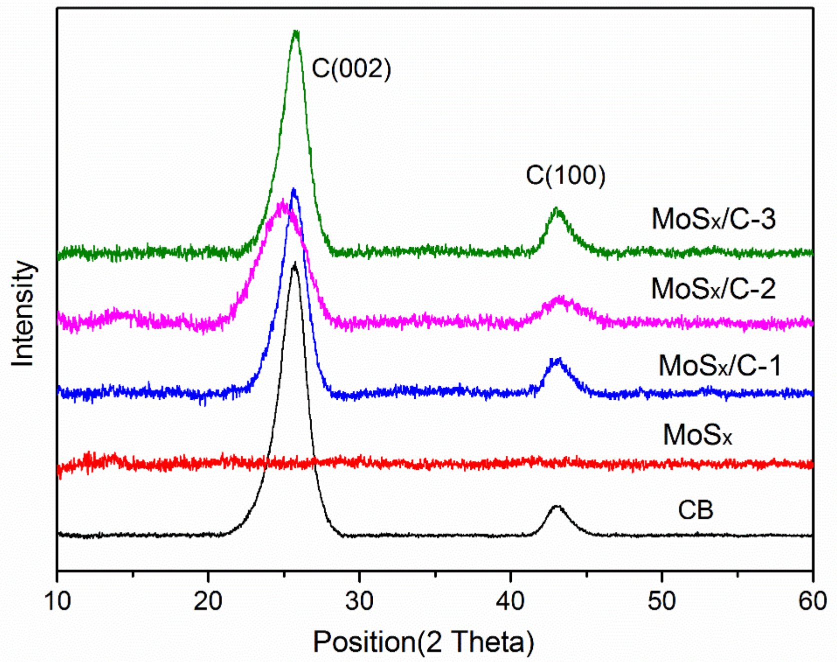

Figure S1: XRD patterns of CB, MoS_x and MoS_x/C nanocomposites.

Table S1: HER performance of different MoS_x-based catalysts

Catalysts	Structure	Onset η(mV)	η at j = 10 mA cm^{-2}	References
**MoS_2**	2H	100	251	13
**MoS_2:Cu/rGO**	2H-1T	/	182	14
**MoS_x/rGO**	Amorphous	/	157	10
**MoS_x**	Amorphous	/	210	16
**MoS_x**	Amorphous	/	200	17
**MoS_x**	Amorphous	~100	~180	32
**MoS_x/C**	Amorphous	40	76	This work

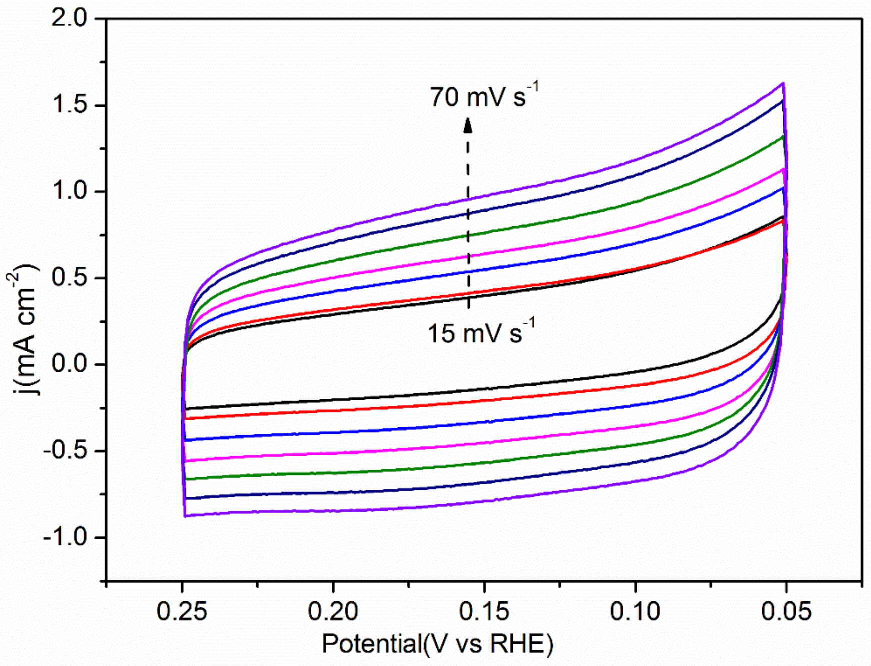

Figure S2: Cyclic voltammetry curves of MoS_x/C-2 at various scan rates (15, 20, 30, 40, 50, 60,70 mV s^{-1}) in the region of 0.25−0.05 V versus RHE.

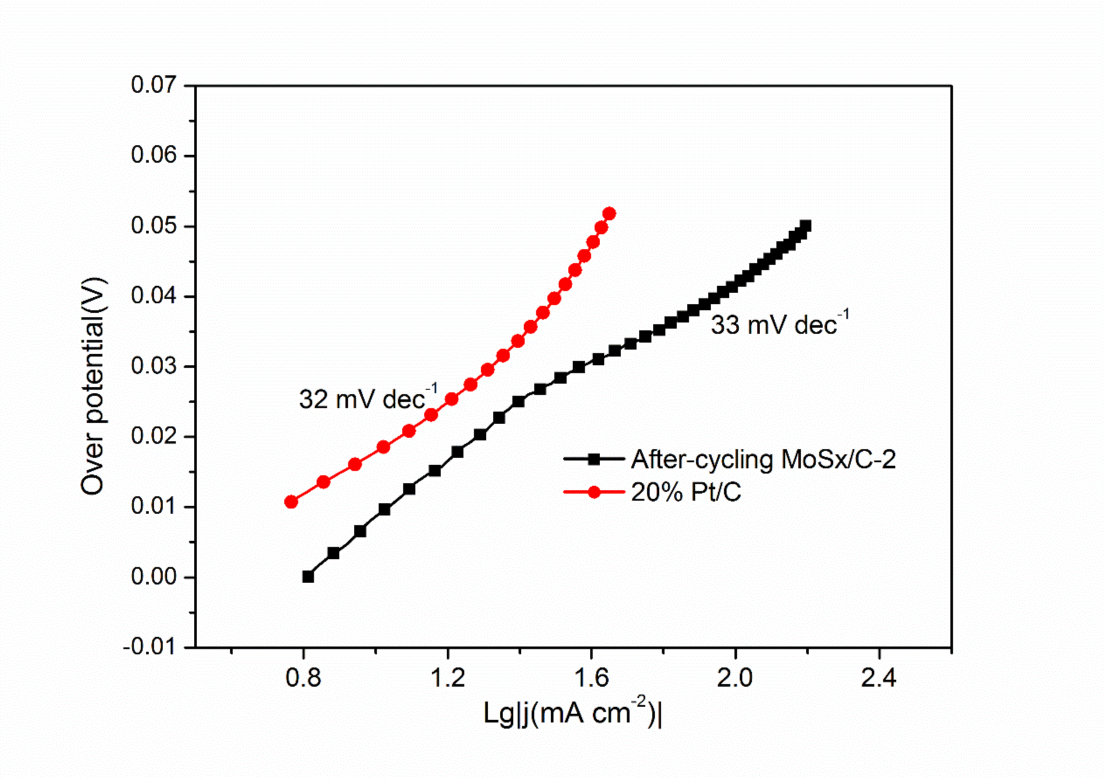

Figure S3: Tafel plots of after-cycling MoS_x/C-2 and 20% Pt/C (Pt foil as counter electrode and MoS_x/C-2 modified carbon paper as working electrode)

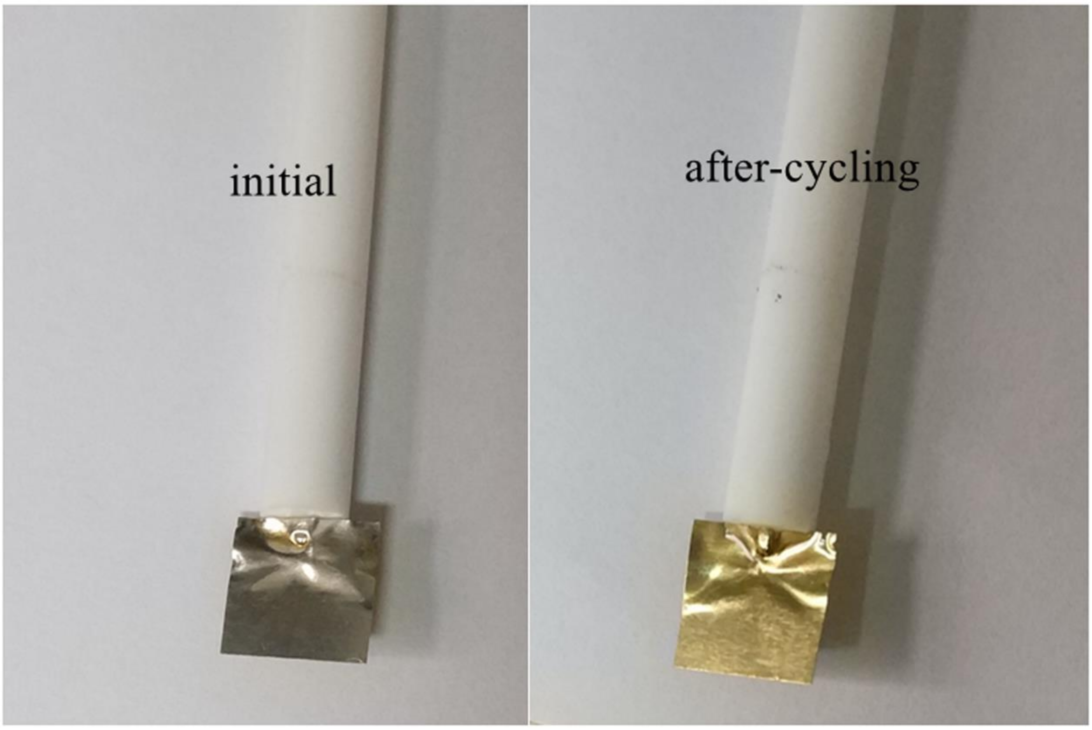

Figure S4: Photographs of Pt counter electrode of initial and after-cycling

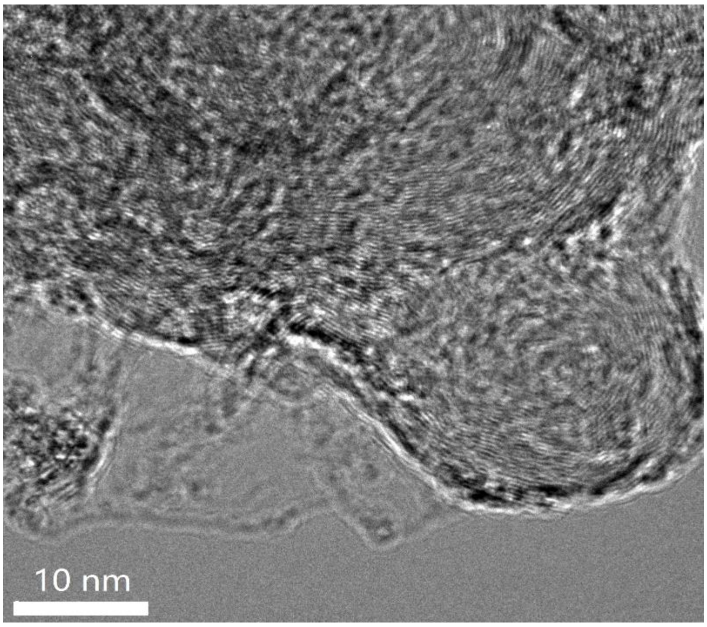

Figure S5: TEM image of after-cycling MoS_x/C-2(Graphite rod as counter electrode)

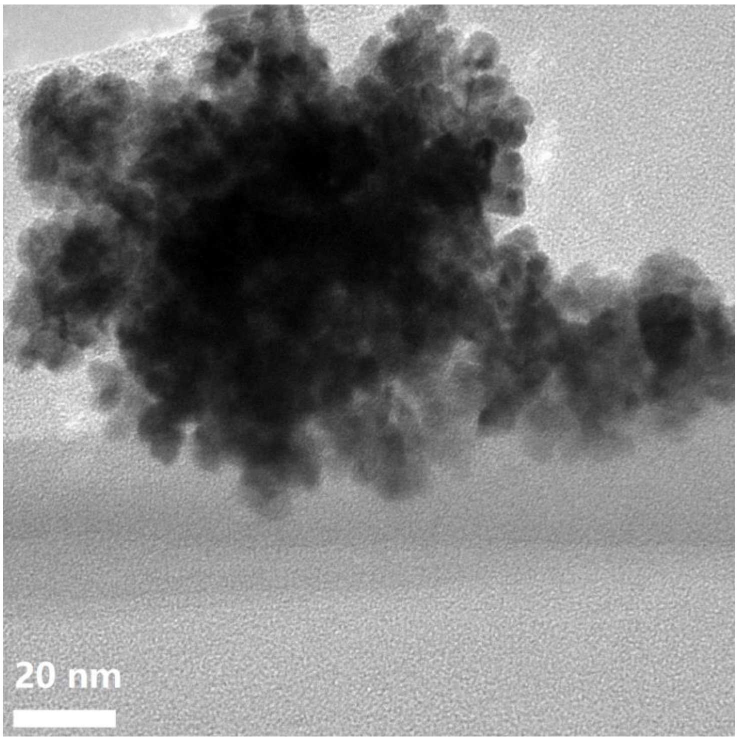

Figure S6: TEM image of Pt aggregation of after-cycling MoS_x/C-2

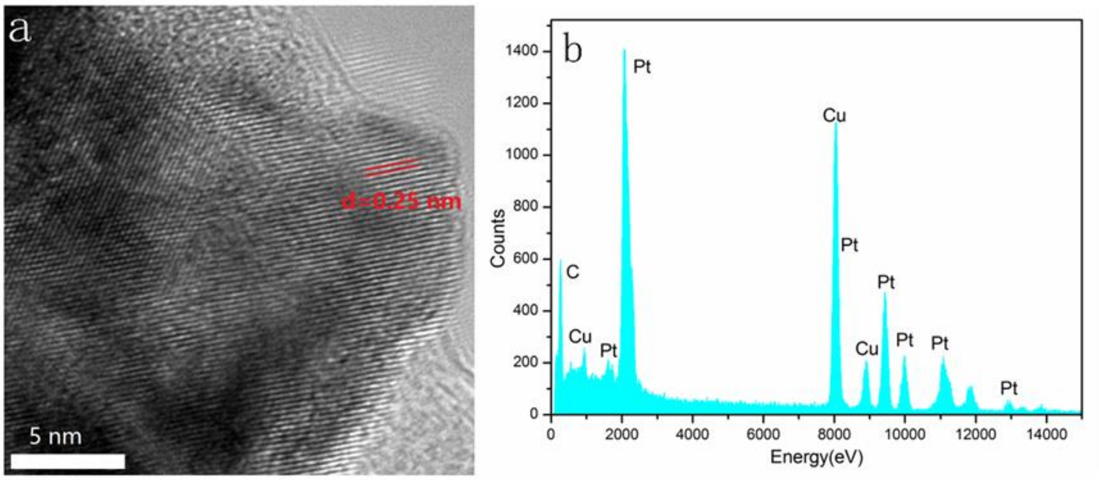

Figure S7: HRTEM image of after-cycling MoS_x/C-2 (a)and corresponding EDS spectrum(b).

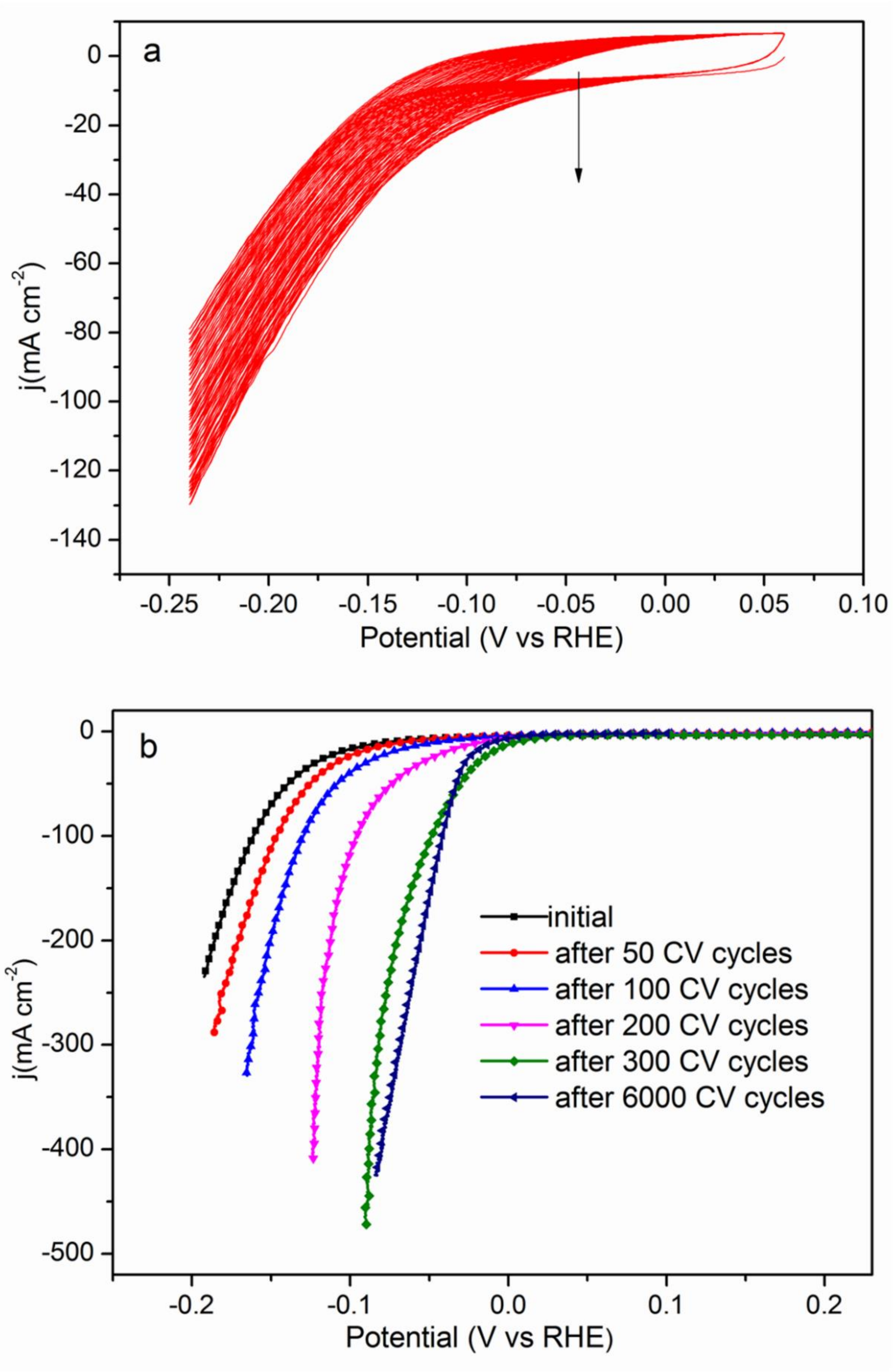

Figure S8: CV and LSV tests of MoS_x/C-2. (a) First 50 CV tests of MoS_x/C-2(scan rate:50 mV s^{-1}); (b) LSV tests of MoS_x/C-2 after different CV cycles.
